# High-spin iron(II) complexes with mono-phosphorylated 2,6-diaminopyridine ligands

**DOI:** 10.1007/s00706-016-1731-9

**Published:** 2016-03-30

**Authors:** Christan Schröder-Holzhacker, Berthold Stöger, Ernst Pittenauer, Günther Allmaier, Luis F. Veiros, Karl Kirchner

**Affiliations:** 1 Institute of Applied Synthetic Chemistry, Vienna University of Technology, Getreidemarkt 9/163-OC, 1060 Vienna, Austria; 2 Institute of Chemical Technologies and Analytics, Vienna University of Technology, Getreidemarkt 9, 1060 Vienna, Austria; 3 Centro de Química Estrutural, Instituto Superior Técnico, Universidade de Lisboa, Av. Rovisco Pais No. 1, 1049-001 Lisbon, Portugal

**Keywords:** Iron, Aminophosphine ligands, DFT calculations, Carbon monoxide

## Abstract

**Abstract:**

Several new monophosphorylated 2,6-diaminopyridine ligands bearing P*i*Pr_2_ and P*t*Bu_2_ units (PN^NH2^-*i*Pr, PN^NH2^-*t*Bu, PN^NHMe^-*i*Pr, and PN^NHEt^-*i*Pr) are prepared by treatment of the respective 2,6-diaminopyridines with the chlorophosphines P*i*Pr_2_Cl and P*t*Bu_2_Cl in the presence of a base. Treatment of anhydrous FeCl_2_ with 1 equiv of these afforded the tetracoordinated coordinatively unsaturated 14e^−^ complexes [Fe(κ^2^*P,N*-PN^NH2^-*i*Pr)Cl_2_] and [Fe(κ^2^*P,N*-PN^NH2^-*t*Bu)Cl_2_], while with PN^NHMe^-*i*Pr and PN^NHEt^-*i*Pr a phosphine transfer reaction of a second PN ligand took place to yield the known PNP pincer complexes [Fe(κ^3^*P,N,P*-PNP^Me^-*i*Pr)Cl_2_] and [Fe(κ^3^*P,N,P*-PNP^Et^-*i*Pr)Cl_2_]. The four-coordinate complexes [Fe(κ^2^*P,N*-PN^NH2^-*i*Pr)Cl_2_] and [Fe(κ^2^*P,N*-PN^NH2^-*t*Bu)Cl_2_] did not react with CO and the formation of iron PNC pincer complexes was not observed. The reason for the reluctance to add CO was investigated in detail by DFT calculations.

**Graphical abstract:**

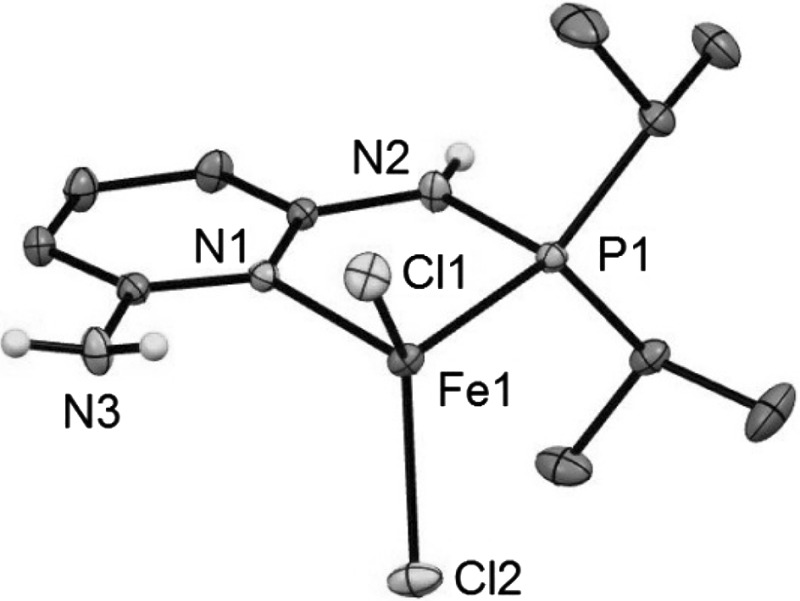

## Introduction

Among the many ligand systems that can be found in the chemical literature, pincer ligands play an important role and their complexes have attracted tremendous interest due to their high stability, activity, and variability [1–5]. Pincer ligands are often planar scaffolds consisting of an anionic or neutral central aromatic backbone tethered to two, mostly bulky, two-electron donor groups by different spacers where steric, electronic, and stereochemical parameters can be manipulated by modifications of the substituents at the donor sites and/or the spacers. Phosphine-based PCP and PNP type ligands having central C and N donors have received the most attention.

In the present contribution we aimed at an in situ synthesis of iron complexes with a new type of pincer ligand, namely a PNC pincer ligand, where the pyridine backbone is connected to an aminophosphine and a carbamoyl moiety (Scheme 1). Obviously, the prerequisite for these reactions is strong coordination of CO to the metal center [6]. The carbamoyl moieties may be formed via an intramolecular attack of the free amine substituent at an electrophilic coordinated CO. It has to be noted that the formation of carbamoyl ligands was already reported by the reaction of rhenium and ruthenium carbonyl complexes with amine-substituted nitrogen-containing heterocycles [7–10]. Moreover, this approach was also adapted to synthesize ferracyclic carbamoyl structures [11, 12].

**Figure:**
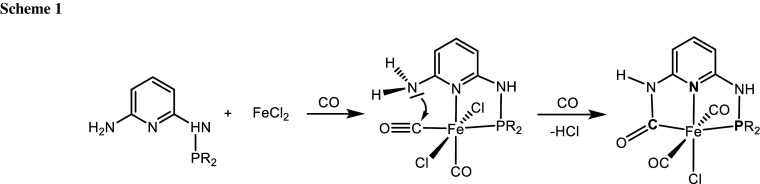

We describe here reactions of mono-phosphorylated 2,6-diaminopyridine ligands with FeCl_2_ in the presence of CO as well reactions with Fe(CO)_4_Br_2_. In the latter, CO is already coordinated to the metal center. Mechanistic studies, based on DFT calculations, dealing with the coordination of CO are also presented.

## Results and discussion

The new PN ligands PN^NH2^-*i*Pr (**1a**), PN^NH2^-*t*Bu (**1b**), PN^NHMe^-*i*Pr (**1c**), and PN^NHEt^-*i*Pr (**1d**) are prepared conveniently in 45–99 % yield by treatment of the respective 2,6-diaminopyridines with 1 equiv of the chlorophosphines P*i*Pr_2_Cl and P*t*Bu_2_Cl in the presence of a base (NEt_3_ or *n*-BuLi) (Scheme 2). The crude product had to be purified by flash chromatography to remove unreacted starting material and the doubly phosphorylated by-product PNP-*i*Pr and PNP-*t*Bu [13]. All reactions were carried out in toluene or toluene/THF at temperatures between 25 and 90 °C for 15 h. The ligands were isolated as air stable solids or oils and were characterized by elemental analysis, ^1^H, ^13^C{^1^H}, and ^31^P{^1^H} NMR spectroscopy. Most diagnostic is the ^31^P{^1^H} NMR spectrum exhibiting a singlet at 47.4, 58.2, 70.0, and 78.8 ppm for **1a**–**1d**, respectively. In the ^1^H NMR spectrum the NH_2_ and NH protons give rise to a slightly broadened singlet in the range of 3.02–4.19 ppm, while the NH*i*Pr_2_ NH*t*Bu_2_ protons in **1a** and **1b** exhibit doublets at 4.40 and 4.67 ppm, with *J*_*HP*_ coupling constants of 10.7 and 11.0 Hz, respectively. All other resonances are unremarkable and are not discussed here.

**Figure:**
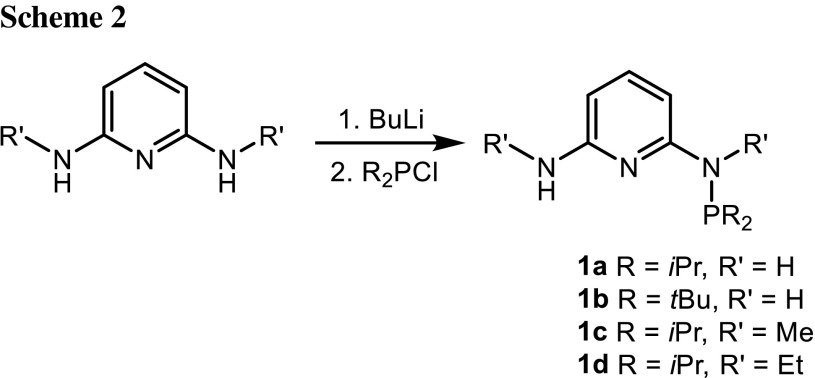

Treatment of anhydrous FeCl_2_ with 1 equiv of the PN ligands PN-*i*Pr (**1a**) and PN-*t*Bu (**1b**) in THF at room temperature afforded the tetracoordinated coordinatively unsaturated 14e^−^ complexes [Fe(κ^2^*P,N*-PN^NH2^-*i*Pr)Cl_2_] (**2a**) and [Fe(κ^2^*P,N*-PN^NH2^-*t*Bu)Cl_2_] (**2b**) in 79 and 81 % isolated yields (Scheme 3). These complexes are air sensitive both in the solid state and in solution and are poorly soluble in most common solvents. They display large paramagnetic shifted ^1^H NMR spectra. At room temperature the line widths are relatively narrow and in the case of **2a** the proton resonances could be readily assigned on the basis of integration. The isopropyl methyl hydrogen atoms appear at 16.2 (6H) and −3.4 ppm (6H), the CH protons give rise to a signal at 150.7 ppm (2H), whereas the pyridine hydrogen atoms are centered at 54.4 (1H), 45.7 (1H), and −19.7 ppm (1H). The NH and NH_2_ protons could not be detected (Scheme 3).

**Figure:**
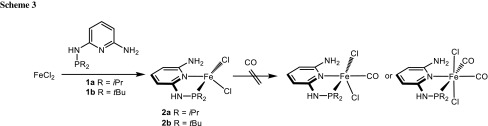

Since ESI–MS enables not only the detection and the study of reaction substrates and products but also short-lived reaction intermediates and decomposition products as they are present in solution, complex **2a** was investigated by means of this technique. A methanolic solution of **2a** was subjected to ESI–MS analysis in the positive ion mode.

Under the so-called “soft ionization” conditions in the electrospray source, the ESI mass spectrum of [Fe(κ^2^*P,N*-PN^NH2^-*i*Pr)Cl_2_] (**2a**) shows prominent peaks at *m*/*z* = 541.2 and 226.1 assignable to the mononuclear species [Fe(κ^2^*P,N*-PN^NH2^-*i*Pr)_2_Cl]^+^ ([M + PN-Cl]^+^) together with the protonated PN^NH2^-*i*Pr ligand (**1a**), respectively. The formation of [Fe(κ^2^*P,N*-PN^NH2^-*i*Pr)_2_Cl]^+^ is unexpected as this compound contains two PN^NH2^-*i*Pr ligands. The fragmentation of the selected [Fe(PN^NH2^-*i*Pr)_2_Cl]^+^ ion with *m*/*z* = 541.2 by low energy collision-induced dissociation (CID) in an ion trap analyzer resulted in the formation of an ion with *m*/*z* = 316.0 due to the loss of a PN^NH2^-*i*Pr ligand (Fig. 2). Cationic pentacoordinate [Fe(κ^2^*P,N*-PN^R^-*i*Pr)_2_Cl]^+^ complexes bearing two PN ligands could not be prepared, despite the fact that these species were the most prominent fragment in the ESI MS spectrum.

**Fig. 1:**
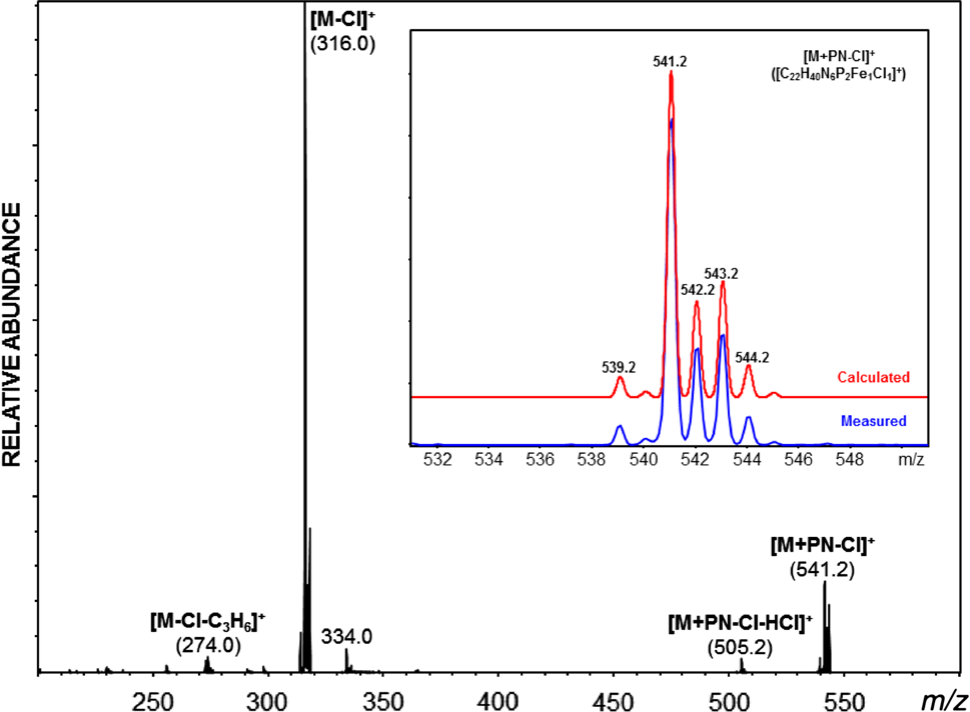
MS/MS (low energy CID)-spectrum of in-source-generated [Fe(κ^2^ *P,N*-PN^NH2^-*i*Pr)_2_Cl]^+^ ([M + PN − Cl]^+^) (*m/z* = 541.2) precursor ions in CH_3_OH. Inset shows the calculated and measured isotopic pattern of the cation [Fe(κ^2^ *P,N*-PN^NH2^-*i*Pr)_2_Cl]^+^ ([M + PN − Cl]^+^). All mass calculations and mass assignments are based on the most abundant iron isotope ^56^Fe and the Cl isotope of lowest mass (^35^Cl)

**Fig. 2:**
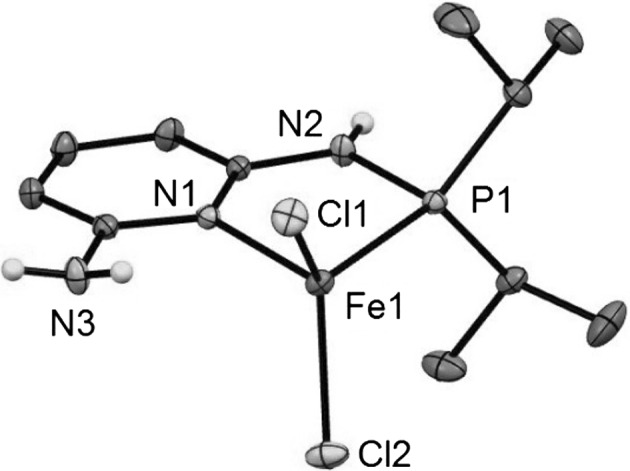
Structural view of [Fe(κ^2^ *P,N*-PN^NH2^-*i*Pr)Cl_2_] (**2a**) showing 50 % thermal ellipsoids (most H atoms omitted for clarity). Only one of the two crystallographically independent complexes is shown. Selected bond lengths (Å) and bond angles (°): Fe1–Cl1 2.2740(4), Fe1–Cl2 2.2369(4), Fe1–P1 2.4038(5), Fe1–N1 2.106(1), P1–N2 1.690(1), Cl1–Fe1–Cl2 118.81(2), Cl1–Fe1–P1 112.18(2), Cl1–Fe1–N1 111.19(3), Cl2–Fe1–P1 119.91, Cl2–Fe1–N1 106.74, Cl1–Fe1–P1–N2 104.95(5), Cl2–Fe1–P1

In addition, the structure of complex **2a** was determined by X-ray crystallography. The molecular structure of **2a** is depicted in Fig. 1 with selected bond distances and angles given in the caption. The structure of the four-coordinate complexes [Fe(κ^2^*P*,*N*-PN^NH2^-*i*Pr)Cl_2_] (**2a**) shows a distorted tetrahedral coordination environment around the iron center. All bond lengths are consistent with a high-spin electron configuration of Fe^2+^ and in reasonable accord with other crystallographically characterized four-coordinate Fe(II) dihalide complexes featuring aminophosphine co-ligands [13–16] (Fig. 2).

On the other hand, complexation was unsuccessful with the ligand PN^NHMe^-*i*Pr (**1c**) and PN^NHEt^-*t*Bu (**1d**). Instead, the reaction of FeCl_2_ with **1c** and **1d** was accompanied by a phosphine transfer step by a second PN ligand to yield the known complexes [Fe(κ^3^*P,N,P*-PNP^Me^-*i*Pr)Cl_2_] (**3a**) and [Fe(κ^3^*P,N,P*-PNP^Et^-*i*Pr)Cl_2_] (**3b**) [16] together with the 2,6-diaminopyridines **4a** and **4b** as well as intractable iron compounds (Scheme 4). Accordingly, the yields of **3a** and **3b** are less than 50 % being 46 and 48 %, respectively. The products were identified by ^1^H NMR spectroscopy, after the insoluble inorganic residue was removed by filtration and comparison with the spectra of authentic samples prepared independently [16].

**Figure:**
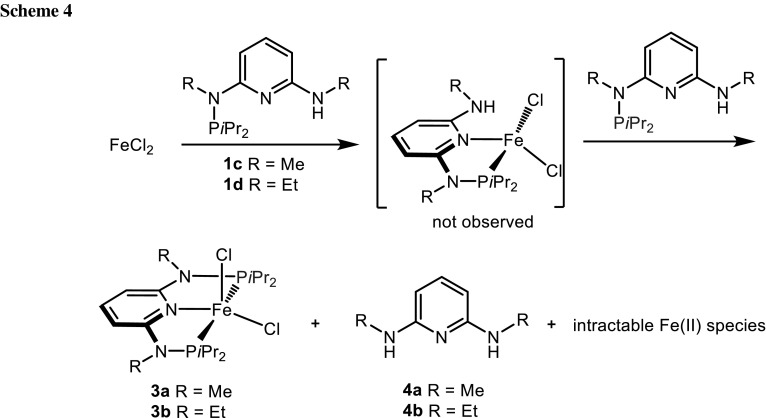

**Fig. 3:**
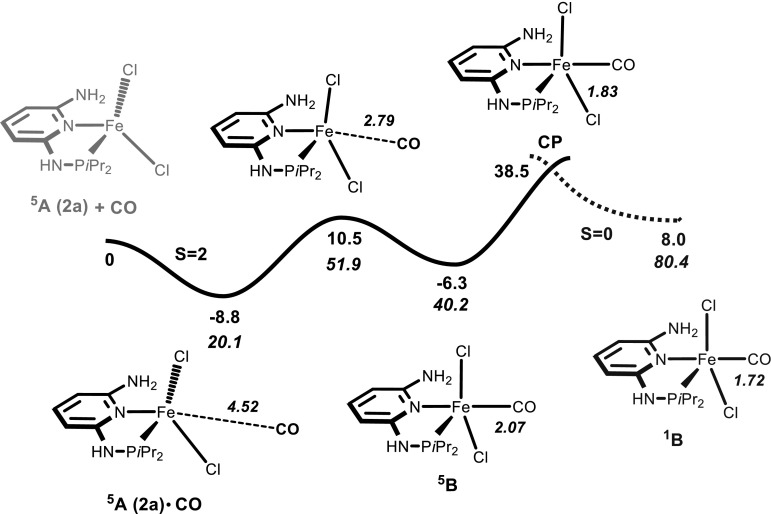
Energy profile (OPBE) for the addition of CO to [Fe(κ^2^ *P,N*-PNP-*i*Pr)Cl_2_] (**2a**) yielding the illusive complex [Fe(κ^2^ *P,N*-PNP-*i*Pr)(CO)Cl_2_] (**B**). The energy values (kJ/mol) are relative to the separated reagents, and the values in italic represent the free energies. The *plain curve* corresponds to the spin-quintuplet PES (*S* = 2), and the *dashed curve* to the spin-singlet PES (*S* = 0). The Fe–C(CO) distance (Å) along the reaction coordinate is indicated

The coordinatively unsaturated complexes [Fe(κ^2^*P,N*-PN^NH2^-*i*Pr)Cl_2_] (**2a**) and [Fe(κ^2^*P*,*N*-PN^NH2^-*t*Bu)Cl_2_] (**2b**) where treated with CO but failed to react (Scheme 3). With Fe(CO)_4_Br_2_ no clean reaction took place and several intractable materials were formed. To rationalize why these complexes do not reacted with CO, the addition of CO to **2a** (denoted as ^**5**^**A** in Fig. 3) was investigated by means of DFT calculations. While ^**5**^**A** has a spin-quintet (*S* = 2) ground state, the mono carbonyl complex [Fe(κ^2^*P,N*-PN^NH2^-*i*Pr)(CO)Cl_2_] (**B**) may exist either as spin-quintet or a spin-singlet (*S* = 0). The energy profile associated with such a reaction goes through a minimum-energy crossing point (MECP) of the two potential energy surfaces (PES) involved [17]. Once that point is reached, there is a given probability for the system to change spin state and hop from one PES to the other and, thus, give rise to a “spin-forbidden” or “non-adiabatic” reaction [18].

Starting with the separated reactants and following the *S* = 2 PES, there is formation of a van der Waals pair between the two reacting molecules ^**5**^**A**·**CO** with a rather long Fe–C(CO) distance (4.52 Å), and the corresponding small stabilization of the system (Δ*E* = −8.8 kJ/mol). From here, the high spin isomer of the product (^**5**^**B**) is formed in a single step going over an accessible energy barrier (Δ*E*^#^ = 19.3 kJ/mol). The formation of ^**5**^**B** is lightly endergonic with Δ*E* = 2.5 kJ/mol, showing that intermediate ^**5**^**B** is slightly less stable than the corresponding pair of reactants. After the formation of the high spin CO adduct ^**5**^**B**, the strongly π-accepting ligand CO may promote a spin change to form the corresponding low-spin complex ^**1**^**B** (*S* = 0). The MECP between the two potential energy surfaces (**CP**) is easily reached, with an associated energy barrier of Δ*E* = 46.9 kJ/mol. Once the crossing point **CP** is reached and the hopping between surfaces is accomplished, the system follows the *S* = 0 PES downhill until the formation of the low spin ^**1**^**B**. However, this process is unfavorable with the putative low-spin product ^**1**^**B** being 14.3  kJ/mol less stable than ^**5**^**B** and less stable by 8.0 kJ/mol with respect to the initial separated reagents ^**5**^**A** and CO. Importantly, the overall balance for the reactions considering free energy values (values in italics in Fig. 3), indicate that CO addition to ^**5**^**A** is clearly endergonic (Δ*G* = 40.2 kJ/mol for ^**5**^**B** and even 80.4 kJ/mol for ^**1**^**B**). Also the formation of an illusive dicarbonyl complex [Fe(κ^2^*P,N*-PN^NH2^-*i*Pr)(CO)_2_Cl_2_] (**C**) (not shown in Fig. 3) is endergonic by 43.5 kJ/mol. This indicates thermodynamically unfavorable processes being in good accordance with the experimental results, since formation of CO adducts were not observed.

In conclusion, we describe here the synthesis of several new monophosphorylated 2,6-diaminopyridine ligands bearing P*i*Pr_2_ and P*t*Bu_2_ units (PN^NH2^-*i*Pr, PN^NH2^-*t*Bu, PN^NHMe^-*i*Pr, and PN^NHEt^-*i*Pr). These ligands react with anhydrous FeCl_2_ to afforded the coordinatively unsaturated paramagnetic complexes [Fe(κ^2^*P*,*N*-PN^NH2^-*i*Pr)Cl_2_] and [κ^2^*P*,*N*-Fe(PN^NH2^-*t*Bu)Cl_2_], while with PN^NHMe^-*i*Pr and PN^NHEt^-*i*Pr a phosphine transfer reaction of a second PN ligand took place to yield the known PNP pincer complexes [Fe(κ^3^*P*,*N*,*P*-PNP^Me^-*i*Pr)Cl_2_] and [Fe(κ^3^*P*,*N*,*P*-PNP^Et^-*i*Pr)Cl_2_]. The four-coordinate complexes did not react with CO and formation of iron PNC pincer complexes was not observed. The reason for the reluctance to add CO was investigated in detail by DFT calculations indicating a thermodynamically unfavorable process.

## Experimental

All manipulations were performed under an inert atmosphere of argon using Schlenk techniques or in an MBraun inert-gas glovebox. The solvents were purified according to standard procedures [19]. The ligands *N*^2^-(diisopropylphosphanyl)pyridine-2,6-diamine (PN^NH2^-*i*Pr) (**1a**), *N*^2^-(di-*tert*-butylphosphanyl)pyridine-2,6-diamine (PN^NH2^-*t*Bu) (**1b**) were prepared according to the literature [15]. The deuterated solvents were purchased from Aldrich and dried over 4 Å molecular sieves. ^1^H, ^13^C{^1^H}, and ^31^P{^1^H} NMR spectra were recorded on Bruker AVANCE-250, AVANCE-300 DPX, and AVANCE-400 spectrometers. ^1^H and ^13^C{^1^H} NMR spectra were referenced internally to residual protio-solvent, and solvent resonances, respectively, and are reported relative to tetramethylsilane (*δ* = 0 ppm). ^31^P{^1^H} NMR spectra were referenced externally to H_3_PO_4_ (85 %) (*δ* = 0 ppm).

All mass spectrometric measurements were performed on an Esquire 3000^*plus*^ 3D-quadrupole ion trap mass spectrometer (Bruker Daltonics, Bremen, Germany) in positive-ion mode by means of electrospray ionization (ESI). Mass calibration was done with a commercial mixture of perfluorinated trialkyl-triazines (ESI Tuning Mix, Agilent Technologies, Santa Clara, CA, USA). All analytes were dissolved in methanol “hypergrade for LC–MS Lichrosolv” quality (Merck, Darmstadt, Germany) to form a concentration of roughly 1 mg/cm^3^. Direct infusion experiments were carried out using a Cole Parmer model 74900 syringe pump (Cole Parmer Instruments, Vernon Hills, IL, USA) at a flow rate of 2 mm^3^/min. Full scan and MS/MS (low energy CID)-scans were measured in the *m/z* range 100–1100 with the target mass set to *m/z* = 1000. Further experimental conditions include: drying gas temperature: 150 °C; capillary voltage: −4 kV; skimmer voltage: 40 V; octapole and lens voltages: according to the target mass set. All mass calculations are based on the most abundant metal isotope ^56^Fe and the Cl isotope of lowest mass (^35^Cl). Mass spectra were averaged during data acquisition time of 1–2 min and one analytical scan consisted of five successive micro scans resulting in 50 and 100 analytical scans, respectively, for the final full scan mass spectrum.

### *N*^2^-(Diisopropylphosphanyl)-*N*^2^,*N*^6^-dimethylpyridine-2,6-amine (PN^Me,NMe^-iPr) (**1c**, C_12_H_22_N_3_P)

*N*^*2*^*,N*^*6*^-Dimethylpyridine-2,6-diamine (22.96 mmol, 3.15 g) was dissolved in 100 cm^3^ toluene and cooled to 0 °C. *n*-BuLi (24.11 mmol, 2.5 M, 9.6 cm^3^) was added and the reaction was stirred at room temperature for 2 h. After cooling to 0 °C, 3.50 g P*i*Pr_2_Cl (22.96 mmol) was added and the mixture was stirred at 80 °C for 12 h. The reaction was quenched at room temperature by addition of 25 cm^3^ saturated NaHCO_3_ solution, the organic phase was dried over Na_2_SO_4_, filtered and the solvent was removed under reduced pressure. The product was used without further purification for the next step. Yield: 5.26 g (90 %) yellow oil. ^1^H NMR (CDCl_3_, 20 °C): *δ* = 7.22 (t, ^*3*^*J*_*HH*_ = 7.7 Hz, 1H, py^4^), 6.62 (bs, 1H, py^3^), 5.70 (d, ^*3*^*J*_*HH*_ = 8.0 Hz, 1H, py^5^), 4.27 (s, 1H, N*H*), 3.02 (s, 3H, N(H)C*H*_*3*_), 2.82 (d, ^*3*^*J*_*PH*_ = 5.1 Hz, 3H, N(P)C*H*_*3*_), 2.21 (m, 2H, C*H*(CH_3_)_2_), 1.08 (dd, ^*3*^*J*_*PH*_ = 17.0 Hz, ^*3*^*J*_*HH*_ = 7.0 Hz, 6H, CH(C*H*_*3*_)_2_), 0.97 (dd, ^*3*^*J*_*PH*_ = 12.1 Hz, ^*3*^*J*_*HH*_ = 7.0 Hz, 6H, CH(C*H*_*3*_)_2_) ppm; ^13^C{^1^H} NMR (CDCl_3_, 20 °C): *δ* = 160.45 (d, ^*2*^*J*_*CP*_ = 20.2 Hz, py^2^), 158.94 (s, py^6^), 137.07 (s, py^4^), 99.37 (d, ^*3*^*J*_*CP*_ = 21.4 Hz, py^3^), 93.99 (s, py^5^), 33.80 (bs, *C*H(CH_3_)_2_), 29.12 (s, N(H)*C*H_3_), 26.21 (d, ^*2*^*J*_*CP*_ = 14.6 Hz, N(P)*C*H_3_), 19.68 (s, CH(*C*H_3_)_2_), 19.40 (d, ^*2*^*J*_*CP*_ = 12.7 Hz, CH(*C*H_3_)_2_) ppm; ^31^P{^1^H} NMR (CDCl_3_, 20 °C): *δ* = 70.0 ppm.

### *N*^2^-(Diisopropylphosphanyl)-*N*^2^,*N*^6^-diethylpyridine-2,6-amine (PN^Et,NEt^-iPr (**1d**, C_13_H_24_N_3_P)

*N*^*2*^*,N*^*6*^-Diethylpyridine-2,6-diamine (121.60 mmol, 3.75 g) was dissolved in 200 cm^3^ toluene and 9.1 cm^3^*n*-BuLi (22.69 mmol, 2.5 M) was added at 0 °C. After stirring at room temperature for 2 h, the mixture was cooled to 0 °C and 3.30 g P*i*Pr_2_Cl (21.60 mmol) was added. The reaction was stirred at 80 °C for 12 h. After quenching with 25 cm^3^ of a saturated NaHCO_3_ solution, the organic phase was dried over Na_2_SO_4_, filtered and concentrated. The resulting yellow oil was used directly without further purification for subsequent reactions. Yield: quantitative, yellow oil. ^1^H NMR (CDCl_3_, 20 °C): *δ* = 7.20 (m, 1H, py^4^), 6.47 (bs, 1H, py^3^), 5.67 (d, ^*3*^*J*_*HH*_ = 7.9 Hz, 1H, py^5^), 4.14 (s, 1H, N*H*), 3.62 (m, 2H, N(P)C*H*_2_CH_3_), 3.19 (m, 2H, N(H)C*H*_2_CH_3_), 2.29 (m, 2H, C*H*(CH_3_)_2_), 1.13–0.82 (m, 18H, CH_2_C*H*_3_, CH(C*H*_3_)_2_) ppm; ^13^C{^1^H} NMR (CDCl_3_, 20 °C): *δ* = 159.11 (bs, py^2^), 158.26 (s, py^6^), 139.05 (s, py^4^), 102.04 (bs, py^3^), 94.28 (s, py^5^), 42.82 (bs, N(P)*C*H_2_CH_3_), 36.90 (s, N(H)*C*H_2_CH_3_), 26.20 (d, ^*1*^*J*_*CP*_ = 15.1 Hz, *C*H(CH_3_)_2_), 19.87 (d, ^*2*^*J*_*CP*_ = 10.0 Hz, CH(*C*H_3_)_2_), 19.39 (s, CH(*C*H_3_)_2_), 14.93 (s, N(H)CH_2_*C*H_3_), 14.73 (s, N(P)CH_2_*C*H_3_) ppm; ^31^P{^1^H} NMR (CDCl_3_, 20 °C): *δ* = 78.8 ppm.

### [Dichloro)(*N*^2^-(diisopropylphosphanyl)pyridine-2,6-diamine)iron(II)] ([Fe(κ^2^P,N-PN^NH2^-iPr)Cl_2_]) (**2a**, C_11_H_20_Cl_2_FeN_3_P)

Ligand **1a** (11.33 mmol, 300 mg) was stirred with 161 mg anhydrous FeCl_2_ (1.27 mmol) in 15 cm^3^ THF for 12 h. The yellow suspension was concentrated to 0.5 cm^3^ and the product was precipitated with 40 cm^3^ Et_2_O. After filtration, the yellow solid was washed twice with 10 cm^3^ of Et_2_O and dried under vacuum. Yield: 352 mg (79 %) as yellow solid. ^1^H NMR (acetone-*d*_6_, 20 °C): *δ* = 150.7 (2H, C*H*(CH_3_)_2_), 54.4 (1H, py), 45.7 (1H, py), 16.2 (6H, CH(C*H*_*3*_)_2_, −3.4 (6H, CH(C*H*_*3*_)_2_, −19.7 (1H, py) ppm. NH and NH_2_ resonances could not be detected.

### [Dichloro)(*N*^2^-(di-tert-butylphosphanyl)pyridine-2,6-diamine)iron(II)] ([Fe(κ^2^P,N-PN^NH2^-tBu)Cl_2_]) (**2b**, C_13_H_24_Cl_2_FeN_3_P)

This complex was prepared analogously to **2a** with 300 mg **1b** (1.18 mmol) and 143 mg anhydrous FeCl_2_ (1.13 mmol) as starting materials. Yield: 349 mg (81 %) as yellow solid.

### [Dichloro)(*N*^2^,*N*^6^-Bis(diisopropylphosphanyl)-*N*^2^,*N*^6^-dimethylpyridine-2,6-diamine)iron(II)] ([Fe(κ^3^P,N,P-PNP^Me^-iPr)Cl_2_]) (**3a**)

Asuspension of 150 mg anhydrous FeCl_2_ (1.18 mmol) and 300 mg **1c** (1.18 mmol) was stirred in 15 cm^3^ THF at room temperature for 12 h. The solvent was then removed under vacuum and the remaining solid redissolved in 15 cm^3^ CH_2_Cl_2_. Insoluble materials were removed by filtration. The volume of the solution was reduced to about 1 cm^3^ and the product was precipitated by addition of 40 cm^3^*n*-pentane. The yellow solid was collected on a glass frit, washed twice with 10 cm^3^*n*-pentane, and dried under vacuum. Yield: 272 mg (46 %) [16].

### [Dichloro)(*N*^2^,*N*^6^-Bis(diisopropylphosphanyl)-*N*^2^,*N*^6^-diethylpyridine-2,6-diamine)iron(II)] ([Fe(κ^3^P,N,P-PNP^Et^-iPr)Cl_2_]) (**3b**)

This complex was prepared analogously to **3a** with 135 mg anhydrous FeCl_2_ (1.07 mmol) and 300 mg **1d** (1.07 mmol) as starting materials. Yield: 269 mg (48 %) [16].

### X-ray structure determination

X-ray diffraction data of [Fe(κ^2^*P,N*-PN^NH2^-*i*Pr)Cl_2_] (**2a**) (CCDC number 1449666) were collected at *T* = 100 K in a dry stream of nitrogen on a Bruker Kappa APEX II diffractometer system using graphite-monochromatized Mo*K*α radiation (*λ* = 0.71073 Å) and fine sliced *φ*- and *ω***-**scans. Data were reduced to intensity values with SAINT and an absorption correction was applied with the multi-scan approach implemented in SADABS [20]. The structures were solved by charge flipping using SUPERFLIP [21] and refined against *F* with JANA2006 [22]. The non-hydrogen atoms were refined anisotropically. The H atoms connected to C atoms were placed in calculated positions and thereafter refined as riding on the parent atoms. H atoms connected to N were located in difference Fourier maps and the N–H distances restrained to 0.870(1) Å. Molecular graphics were generated with the program MERCURY [23].

### Computational details

Calculations were performed using the Gaussian 09 software package [24], and the OPBE functional [25–28] without symmetry constraints. This functional combines Handy’s OPTX modification of Becke’s exchange functional with the gradient corrected correlation functional of Perdew, Burke, and Ernzerhof, and was shown to be accurate in the calculation of spin state energy splitting for first transition row species and, in particular, for iron complexes [29–31]. The optimized geometries were obtained with the Stuttgart/Dresden ECP (SDD) basis set [32–34] to describe the electrons of the iron atom. For all other atoms a standard 6-31G** basis set was employed [35–40]. Transition state optimizations were performed with the Synchronous Transit-Guided Quasi-Newton Method (STQN) developed by Schlegel et al. [41, 42], following a thorough search of the Potential Energy Surfaces (PES). Frequency calculations were performed to confirm the nature of the stationary points, yielding one imaginary frequency for the transition states and none for the minima. Each transition state was further confirmed by following its vibrational mode downhill on both sides, and obtaining the minima presented on the energy profiles.

The Minimum Energy Crossing Points (MECP) between PES of two different spin states were determined using a code developed by Harvey et al. [43]. This code consists of a set of shell scripts and Fortran programs that use the Gaussian results of energies and gradients of both spin states to produce an effective gradient pointing towards the MECP.

Electronic energy values are presented in the profiles and discussed along the text because MECP are not stationary points and, hence, a standard frequency analysis is not applicable. However, free energy values are also presented for all stationary points, for comparison purposes. Those values were obtained from the electronic energies at 298.15 K and 1 atm using zero point energy and thermal energy corrections based on structural and vibration frequency data and were further corrected for dispersion effects by means of Grimme DFT-D3 method [44] with Becke and Jonhson short distance damping [45–47].
